# Exploring Jordanian women's resistance strategies to domestic violence: A scoping review

**DOI:** 10.3389/fsoc.2022.1026408

**Published:** 2022-11-10

**Authors:** Rula Odeh Alsawalqa, Maissa N. Alrawashdeh, Yara Abdel Rahman Sa'deh, Amal Abuanzeh

**Affiliations:** ^1^Department of Sociology, The University of Jordan, Amman, Jordan; ^2^Department of Sociology and Anthropology, Doha Institute for Graduate Studies (DI), Doha, Qatar; ^3^School of Law, The University of Jordan, Amman, Jordan

**Keywords:** domestic violence, intimate partner violence, resistance strategies, women abuse, Jordanian women

## Abstract

Despite there being an abundant gender and social science research on domestic violence (DV) in Jordan, particularly during the COVID-19 pandemic, there is limited understanding and knowledge of women's resistance strategies to DV. To fill this gap, this study conducted a scoping review to synthesize and analyze 11 articles published in English-language scholarly journals between 2001 and 2021 by following the PRISMA-ScR guidelines. The databases of the University of Jordan Library, Dar Almandumah, PsycINFO, PubMed, Google Scholar, and Scopus were searched in December 2021. Our review found no scientific articles that primarily discussed Jordanian women's resistance to DV and explicate it as a secondary aim within the context of screening for the causes, consequences, and prevalence of DV. Therefore, while a few articles implicitly conceptualized women's resistance in the context of the patriarchal structure—either as tactics of physical, social, economic survival, and to protect their family and honor, or as consequences of DV—no article provided an explicit definition of this concept. The articles also deliberated on 12 resistance strategies that women use to deal with DV; predominant among them are daily resistance, activities hidden for immediate and de facto gains (e.g., to avoid beatings, divorce and family disintegration, the decision to keep their children, and maintaining economic stability). The most common strategies are silence and not seeking help, reporting to family members or friends, seeking legal and social advice, and reporting to the police or healthcare provider.

## Introduction

Domestic violence (DV), as a complex social and power relationship, is a significant social problem and public health issue globally (Perryman and Appleton, 2016), and is considered one of the most common forms of gender-based violence (Flury et al., 2010). The terms “family violence,” “domestic violence,” “violence in the immediate social environment,” and “intimate partner violence” are often used synonymously with DV (Flury et al., 2010; Lambert, 2014; Perryman and Appleton, 2016; Mayo Clinic, 2020). DV is defined as any threatening incident or behavior; violence or abuse; and psychological, emotional, physical, sexual, financial, and harassment occurring within intimate partner relationships or marriages, and can take place in both heterosexual and same-sex relationships (Lambert, 2014). DV may also involve children, parents, grandparents, friends, and teenagers, but the most recurrent occurrence is abuse of women by men (Lambert, 2014; Rogers, 2015; Perryman and Appleton, 2016). The national framework for protecting the family from DV in Jordan defines it as “*Any act or omission that occurs by a family member against any other member of the same family that leads to non-moral or moral harm*,” and includes four types: physical, sexual, or psychological violence, and neglect (National Council for Family Affairs, 2016, p. 7). Women experience serious short-, medium- and long-term negative physical and mental health consequences of DV (Flury et al., 2010; Joyful Heart Foundation, 2022). Moreover, DV can lead to poverty and homelessness (Australian Institute of Health Welfare., 2021).

In Jordan, DV is the most prevalent form of violence against women, and the majority of the victims are wives and children (National Council for Family Affairs, 2008, 2013). The customary DV perpetrators, in particular of physical violence, are the current husband, followed by the former husband, the brother, and father (SIGI-Jordan, 2013; Department of Statistics, Jordan, 2018). In a scoping review study on DV against women in Middle Eastern countries, Kisa et al. (2021) found that the highest prevalence rate was reported in Jordan (98%). Additionally, Jordan recorded the second lowest rates of lifetime violence (50%) after Lebanon (35%). During 2019, 21 cases of domestic killings were recorded—an increase of 300% compared to 2018 which recorded seven murders—and 6,965 female victims of violence (EuroMed Rights, 2020). Furthermore, Jordanian women are arbitrarily detained without charge or trial for a relationship outside marriage (accusation of “zina”) or leaving home without male family members' permission (absence) (Amnesty International, 2019).

During the lockdown which took place between March and May 2020, Jordan saw an increase of 33% in DV, totaling 1,685 cases (932 related to adult women and 753 to children; including 309 cases of males and 440 cases of females), which was handled by social service offices affiliated to the Ministry of Social Development. Additionally, the number of family murders, from the beginning of 2021 to November 23, 2021, were 15 cases. Moreover, the social protection line of the Ministry of Social Development received 1,700 gender-based violence complaints during the lockdown. The protection orders were no longer accessible during the COVID-19 pandemic as the courts were closed, and lawyers were thus unable to represent women seeking legal support but could only provide legal advice and consultation (EuroMed Rights, 2020; SIGI-Jordan, 2020; The Jordanian National Commission for Women, 2020).

DV prevalent in patriarchal societies is a product of unequal power relations between men and women, which assert men's power and dominance over women (Vyas and Jansen, 2018). “Where there is power, there is resistance” (Foucault, 1978, p. 95). Resistance is not outside of power, one is unable to escape it, and its existence is necessary. Power relationships depend on a multiplicity of points of resistance, which are present everywhere in the power network (Malcorps, 2018, p. 40). Jordanian women and girls still face the plight of violence and gender inequality, the effects of a patriarchal system in which men hold primary power and moral authority, and where they incur risks of DV in multiple forms and ways. They realize that living with an abusive partner under the social barriers in Jordan requires enormous strength and adopt hidden or overt successful individual coping strategies to stay safe each day (Scott, 1985; The Jordanian National Commission for Women, 2017; Alsawalqa, 2021a). Resistance exists in messy and dynamic relations (Pain, 2014), whereby women attempt to resist violence, end it and control the harsh conditions they face, especially when abusive men use family, friends, and children to dominate and tighten their control, which may limit women's ability to receive support and help (Bancroft, 2002; Hayes, 2013).

The resistance concept is used to reflect the ability of women to complain, disagree, avoid, and respond to violence and its negative consequences, the abuser, abusive relationships, and environments that foster cultural and social norms of violence against women (Crann and Barata, 2016). This ability is demonstrated in any activity (overt or hidden) through which women attempt to maximize their safety (Paterson, 2009) such as avoidance, help-seeking, active opposition, leaving a violent relationship (Rajah and Osborn, 2020), or fighting back physically (Rajah, 2007; Parker and Gielen, 2014), suicide (Abraham, 2005; Ferraro, 2006). It may be expressed in the form of an intimate practice (Pain, 2014), appeasing and accommodating the husbands and extended families, refraining from requesting money, suppressing their own complaints, and praying and hoping (Critelli, 2012). Among the hidden forms of resistance that women carry out under patriarchal control are bargaining to obtain some gains in exchange for their submission, or secretly saving money, and earning some income by selling the crop without the knowledge of the husband (Benadada et al., 2021, p. 6). During the COVID-19 pandemic, some women resisted DV in various ways: verbal abuse, shouting, threatening divorce, scratching, punching the husband, silent treatment, staying away, stopping communication, not sleeping in the same bed, and refusing to serve the person (Mas'udah et al., 2021).

In Arab and Islamic societies, the majority of women, human rights defenders, and feminists employ legislation in their struggle against gender-based violence. They also resort to different strategies of resistance; most of them take the form of everyday resistance (Qaedabih et al., 2019; Benadada et al., 2021) that Scott (1985, p. 33) described as “informal, covert, and concerned largely with immediate, de facto gains.” Jordan, however, reported the highest proportion of women with no response to DV (Kisa et al., 2021); silence was silence was the most common form of passive response, and women refrained from reporting and submitting formal complaints against perpetrators, despite the existence of legal and institutional protection services (The Jordanian National Commission for Women, 2017; SIGI-Jordan, 2021).

Women's plight has gained the attention of researchers and activists in women's and human rights field from various disciplines such as sociology, gender studies, law and sharia studies, and anthropology. Interestingly, while we examined numerous studies on violence against women, DV, and gender issues in Jordan, and, in particular, while researching the phenomenon of cyber dating violence against women in Jordan, we did not find clear and explicit data about the methods and strategies of resistance that Jordanian women and girls employ against these behaviors. The Arab Council for Social Sciences has issued a book in two issues on “gender resistance” in Arab societies, which includes 17 articles, within the fellowship program “New Paradigms Factory (NPF),” to address gender violence and women's various individual or collective methods of resisting institutional, familial, or societal violence. The first issue (Qaedabih et al., 2019) contained seven articles, and the second issue (Benadada et al., 2021) had 10. Unfortunately, the 17 articles discussed the women's resistance in the majority of Arab societies but did not include the Jordanian society. Nevertheless, there is an abundant scientific production on DV in Jordan. National and international reports, books and articles discuss DV from multiple aspects: its causes, prevalence and forms, the characteristics of battered women and of perpetrators, social services and healthcare for women victims of DV, correlates of DV with demographics and family functioning, the consequences of DV, barriers to identification and treatment of DV, and women's reactions to DV research in Jordan (e.g., Clark et al., 2012). However, little is known about Jordanian women's resistance, and relevant literature comprehensively reviewed is still rare. Therefore, this study aimed to determine, analyze, and fill the knowledge gaps through a scoping review of relevant research. Our review intended to systematically identify and critically review the literature and synthesize the knowledge about the concept, context, and types of Jordanian women's resistance strategies to DV. In light of the increasing cases of DV against women in Jordan, our study was guided by the following questions:

1. How do researchers define the term “resistance” in DV context in Jordan?
2. What methods have been used to explore women's resistance strategies to DV in Jordan?
3. What forms of DV in Jordan that are related with resistance strategies have researchers discussed in their studies?
4. What types of Jordanian women's resistance strategies to DV do researchers discuss in their studies?

## Materials and methods

### Study design

This study's methodology had a qualitative design based on the scoping review approach, also called scoping project, mapping review or literature mapping, systematic mapping, and rapid review (Pham et al., 2014; Peters et al., 2015). Scoping reviews are conducted to identify and analyze knowledge gaps, scope a body of literature, clarify key concepts or definitions, summarize and disseminate research findings and make recommendations for future research (Peters et al., 2015; Munn et al., 2018). Grant and Booth (2009) described scoping review as “preliminary assessment of potential size and scope of available research literature. Aims to identify nature and extent of research evidence (usually including ongoing research)” (p. 95). According to Peters et al. (2015), scoping reviews are “commonly used for ‘reconnaissance,' to clarify working definitions and conceptual boundaries of a topic or field. Scoping reviews are therefore of particular use when a body of literature has not yet been comprehensively reviewed, or exhibits a large, complex, or heterogeneous nature not amenable to a more precise systematic review” (p. 141).

### Sample selection process and data analysis

**Figure 1:**
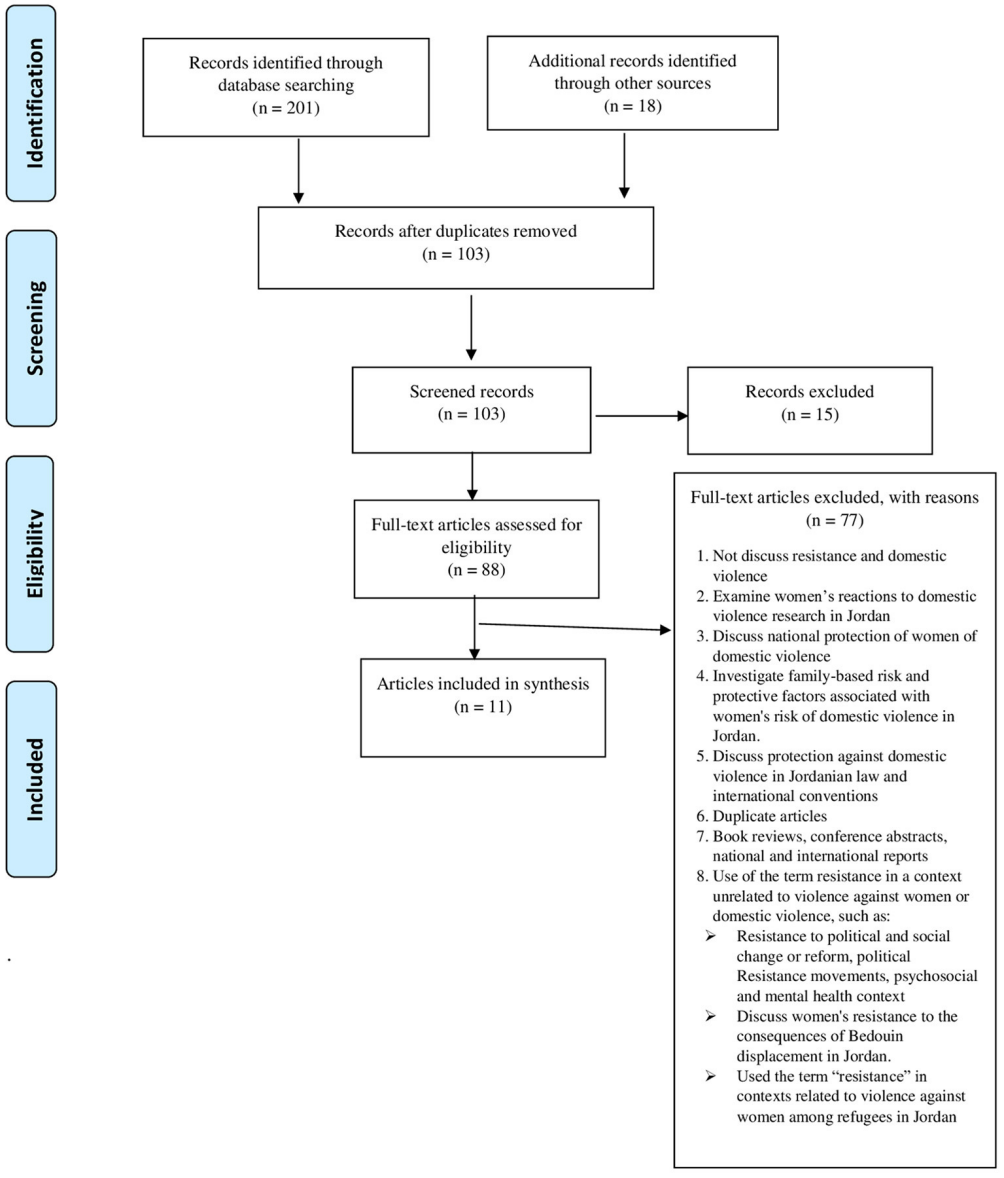
PRISMA-ScR flow diagram.

The scope of this study included only the Hashemite Kingdom of Jordan (Jordanian women, Jordanian families). To this end, we determined our sample of published articles by systematic searches by title, abstract, and keywords in electronic databases (Library University of Jordan, Dar Almandumah, PsycINFO, PubMed, Google Scholar, and Scopus) using four sets of terms: (“Gender-Based violence” + “resistance” + “Jordan,” “partner violence” + “resistance” + “Jordan,” “domestic violence” + “resistance” + “Jordan,” “reaction to abuse,” “response to abuse.” Our scoping review followed the PRISMA-ScR guidelines (Tricco et al., 2018). Figure 1 includes a PRISMA-ScR flow diagram of the steps taken during our sample selection process. The process of our scoping review required engaging with each stage in a reflexive way; no restrictions were specified for relevant articles date, design, and search terms, following the example of Arksey and O'Malley (2005), and Rajah and Osborn (2020).

The preliminary search results varied depending on the search terms used and the databases explored, and showed the severe lack of published studies related to women's resistance to DV in Jordan, so we redefined the search terms and proceeded with more sensitive searches of relevant articles, based on Arksey and O'Malley (2005), with consideration for the fact that researchers used synonymous terms for DV, and the differences between Arab and Jordanian researchers in describing the term “resistance,” such as “prevent,” “response,” and “ending” or “stop violence.” The searches comprised articles published in Arabic and English languages. These initial searches, conducted in December 2021, yielded 201 articles. The search results were exported to EndNote. Articles not closely related to the questions of our study, those using the term “resistance” in a context unrelated to violence against women or DV (such as resistance to political and social change or reform, political resistance movements, psychosocial and mental health context), and duplicate articles, book reviews, conference abstracts, national and international reports, were excluded. Additionally, due to our geographic focus, we also excluded the term “resistance” showing up in contexts related to violence against women among refugees in Jordan. After manual screening and eligibility assessment, 11 articles were identified as the final desired sample. These articles were analyzed based on qualitative coding through deductive approach to extract data for our review. After closely examining and accurately reading the identified articles, familiarizing with the data and taking preliminary notes, we began a process of coding, both preliminary and secondary for created categories: the definition, types of DV, and resistance strategies. Then, we sorted the codes into potential themes to answer the questions of the scoping review, and to reach interpretations that led to research results.

## Results

### Description of studies

The 11 articles included in the final sample were published between 2001 and 2021, in nine separate English-language journals in areas of domestic and partner violence, women's health and psychology. The most frequently represented journals were *Journal of Interpersonal Violence* (two articles), and *Health Care for Women International* (two articles), and *Trauma, Violence, and Abuse* (two articles). The methodology of these studies was qualitative approach (five articles), a majority of which used interviews or personal narratives (three articles), and quantitative (four articles), which were cross-sectional studies (three articles) and prospective cohort ones (four articles). The remaining studies used mixed methods (see Table 1). While one article addressed violence perpetrated by couple partners (Alsawalqa, 2021a), the majority of the selected sample was about violence against married women between 17 and 51 years of age by family members or in-laws; most offenders were husband or ex-husband, father, and brother. The population samples in these studies were from the north, central and southern regions of Jordan, and a few articles took into account the rural and urban classification (such as Haddad et al., 2011); however, most of them chose samples from the central regions, and in particular, the capital, Amman. Moreover, the majority of studies used the terms “intimate partner violence,” “spousal violence,” “wife abuse,” and “wife violence” synonymously with DV and used the term “intimate partner” to refer to the husband.

**Table 1:** Description of final sample (reviewed articles).

**Variable**	*N* (%)^*^
**Language**	
Arabic	0
English	11
**Methodology**	
Qualitative	5 (45.5%)
Interviews/personal narratives	3 (27.3%)
Record keeping	1 (9.1%)
Scoping review	1 (9.1%)
Quantitative	4 (36.4%)
Prospective cohort	1 (9.1%)
Cross-sectional	3 (27.3%)
Mixed methods	2 (18.9%)
**Definition of resistance**	
Explicit	0 (0%)
Implicit	3 (27.3%)
None	8 (72.7%)
**Resistance strategies discussed**	
Not seeking help and silence	7 (63.6%)
Reporting to family members /friends	6 (54.5%)
Reporting to police	4 (36.4%)
Reporting to a religious leader	3 (27.3%)
Bargaining	1 (9.1%)
Reporting to a health-care provider	2 (18.9%)
Locating a shelter	4 (36.4%)
Seeking legal, social advice	5 (45.5%)
Suicide (attempts or thoughts)	4 (36.4%)
Substance use (nicotine, alcohol, painkillers, stimulants, tranquilizers)	2 (18.9%)
Coping behaviors (avoidance, isolation, accepting responsibility and patience, changing her behavior toward her husband)	4 (36.4%)
Divorce/separation	3 (27.3%)
**Forms of domestic violence discussed**	
Physical	10 (90.9%)
Psychological/emotional	7 (63.6%)
Economic	4 (36.4%)
Sexual	10 (90.9%)
Cyber abuse	1 (9.1%)
Control behaviors	5 (45.5%)
Forced marriage	1 (9.1%)
Honor killing	1 (9.1%)

* *N* = 11.

Four main themes have been identified in the reviewed articles: forms of DV, definition of resistance, types of women's resistance strategies to DV, and types of women's resistance strategies vs. forms of DV.

## Findings of the review

### Forms of domestic violence

The results of our scope review revealed eight forms of DV discussed in the context of women's responses and resistance: physical (10 articles), psychological or emotional (seven articles), control behaviors (five articles), economic (four articles), sexual (ten articles), cyber abuse (one article), forced marriage (one article), and honor killing (one article).

### Definitions of resistance

Although all the reviewed articles were not intended primarily to discuss women's resistance to DV or gender resistance, five articles explicitly included the issue of help seeking (especially healthcare), coping strategies, how women deal with violence, and examining women's responses to DV behaviors, as secondary aims within the context of examining DV and its mental health consequences, women's experiences of DV and process of resolution, disclosure of spousal violence, and the role of the extended family in women's risk of DV. However, no article provided an explicit definition of resistance, and eight of them did not define resistance, either implicitly or explicitly. Three papers provided an implicit definition of resistance (see Table 1), where resistance was referred to as the wife's patterns of coping with and response to abuse (Btoush and Haj-Yahia, 2008). In a discussion on women's strategies to cope with electronic dating violence, Alsawalqa (2021a) defined their resistance as an attempt to handle perpetrators' threats and their negative emotional effects through a set of behaviors aimed at preserving their health and psychological wellbeing, and stopping victimization. Safadi et al. (2013) indicated that resistance means women's ability to leave a DV situation to be safe and feel self-worthy and independent, and suggested that women had to feel angry enough to fight back for themselves or their children, and get help (see Table 2). Furthermore, the reviewed articles used multiple terms to describe the term resistance. For example, Btoush and Haj-Yahia (2008) used “coping with wife abuse,” “help strategies,” and “coping methods;” Damra et al. (2015) mentioned “response,” “seeking help;” and Hamdan-Mansour et al. (2012) employed the term “coping strategies.” Two articles explicitly devoted a special section to resistance strategies; one of them used the term “resistance strategies” (Alsawalqa, 2021a), while the other preferred “women's responses to violence” to describe their resistance strategies to DV (Kisa et al., 2021).

**Table 2:** Main evidence: Jordanian women's resistance strategies to domestic violence.

			**Resistance strategies**												**Forms of domestic violence**							
**References**	**Definitions of** **resistance**	**Abuser**	**Did not seek** **help/silence**	**Reported to** **family members/friends/**	**Reported to police**	**Reported to** **religious leader**	**Bargaining**	**Reported to health** **care provider**	**Located shelter**	**Sought legal,** **social advice**	**Suicide**	**Substance use**	**Coping behaviors**	**Divorce/separation**	**Physical**	**Psychological/emotional**	**Economic**	**Sexual**	**Cyber abuse**	**Control behaviors**	**Honor killing**	**Forced marriage**
Al-Modallal (2017)		Husband	✓												✓			✓		✓		
Alsawalqa (2021a)	Implicit female attempts to cope with perpetrators' threats, negative emotional effects, and psychological stress,	Male relative/romantic partner	✓	✓	✓		✓				✓	✓	✓		✓	✓	✓	✓	✓	✓		
Btoush and Haj-Yahia (2008)	Implicit: patterns of coping and response with wife abuse	Husband		✓		✓				✓			✓	✓	✓	✓	✓	✓				
Clark et al. (2010)		Mother-in-law Sister-in-law Father-in-law Brother-in-law Other-in-law Mother, Father Brother, Sister Aunt, Uncle Husband		✓										✓	✓	✓		✓		✓		
Damra et al. (2015)		Current or former husband						✓							✓			✓				
Faqir (2001)		Father Brother							✓		✓										✓	
Haddad et al. (2011)		Husband Ex-husband Brothers	✓												✓	✓		✓				
Hamdan-Mansour et al. (2012)		Intimate Partner Abuse	✓	✓						✓	✓	✓	✓		✓	✓	✓	✓				
Kisa et al. (2021)		Husbands or male partners	✓	✓	✓	✓		✓	✓	✓					✓	✓	✓	✓				
Safadi et al. (2013)	Implicit definition: women ability to leave domestic abuse situation to be safety, and felt of self-worth, independence. For most of the women, in order to leave DV, they had to become angry enough to fight back for themselves or their children, and to coincidentally be offered help	Father, Brother, Husband, Fiancé	✓	✓	✓				✓	✓	✓			✓	✓	✓		✓		✓		✓

Overall, within the context of men's coercive control, and the patriarchal structure that reinforces male supremacy and dominance, the articles conceptualized Jordanian women's resistance to DV from two perspectives: (1) as a tactic of physical, social, economic survival, and to protect their family honor and keep children safe; and (2) as a consequence of DV.

### Types of women's resistance strategies to domestic violence

The 11 articles reported 12 resistance strategies that women use to deal with DV: (1) not seeking help and keeping silence (seven articles); (2) reporting to family members or friends (six articles); (3) reporting to police (four articles); (4) reporting to a religious leader (three articles); (5) bargaining (one article); (6) reporting to a health-care provider (two articles); (7) locating a shelter (four articles); (8) seeking legal and social advice (five articles); (9) suicide (attempts or thoughts) (four articles); (10) substance use: nicotine, alcohol, painkillers, stimulants, tranquilizers (two articles); (11) coping behaviors (avoidance, isolation, accepting responsibility and enduring, changing their behavior toward their husbands) (four articles); and (12) divorce or separation (three articles). Three of them are mainly employed: seeking help or keeping silence (63.6%), reporting to family members or friends (54.5%), and seeking legal, social advice (45.5%). Strategies that they are less used by women are bargaining (9.1%), reporting to a health-care provider (18.9%), substance use (18.9%), reporting to a religious leader (27.3%), and divorce or separation (27.3%) (see Tables 1, 2). The remaining strategies that represent each of them, approximately a third, are mentioned as other options for women to cope with DV: reporting to police (36.4%), suicidal thoughts or attempts (36.4%), and other options for women to cope with DV (avoidance, isolation, accepting responsibility and enduring, changing their behavior toward their husbands) (36.4%), and locating a shelter (36.4%). The percentages of women carrying out resistance strategies varied according to the form of DV they faced, which is explained in the last section of the results (see Table 2).

### Types of women's resistance strategies vs. forms of DV

According to Table 2, the types of resistance strategies intertwine with the eight forms of DV, with a slight variation according to the nature of the violence. Women resort to all twelve strategies (mentioned in Table 1) to resist physical, sexual, psychological or emotional and economic violence. In control situations, they employ all resistance strategies except “reporting to a health-care provider.” They deal with cyber abuse through seven strategies: not seeking help and keeping silence, reporting to family members or friends, reporting to police, bargaining, suicidal thoughts or attempts, substance use, and coping behaviors. Women use only two strategies to deal with attempts at honor killing: locating a shelter and suicide. The majority of the articles discussed DV holistically, with the exception of those that dealt with one or two forms, such as Faqir (2001) who studied honor killing, and Alsawalqa (2021a) who evaluated the problem of electronic dating violence toward women by intimate partners. Alsawalqa does not provide details of the resistance strategies against each form of electronic dating violence explored (e.g., threatening or posting sexual or insulting images and lewd photos, forcing sexual behavior, monitoring, controlling), and against other forms of violence (physical and psychological), despite confirming that the coping strategies varied according to the nature and severity of the abuse, and the differences in the social position of the woman and her educational and economic levels.

Spencer et al. (2014) discussed Jordanian women's non-family help seeking when subjected to physical or sexual violence. They found that women seek help outside the family when the severity of the violence increases, or when the woman's or her husband's family proves unwilling to help (e.g., if the woman was considered to be at fault) or unable to help (e.g., poor economic situation). Nevertheless, sociodemographic characteristics are not closely related with help seeking outside of the family. However, Spencer et al. (2014) pointed out that certain factors play a critical role in limiting the help options, such as financial dependence on the husband's salary, women's educational level, exposure to DV in childhood, number of children, and socio-cultural contexts that justify wife beating. Additionally, despite that family interventions enabled women to save the marital relationship and maintain privacy, it may exposes women to more violence and causes other problems, especially when disclosures of sexual violence are made to family members as it implies refusal to have sex with their husbands and this is thought to bring shame to the entire family; hence, in such a situation, women have no other choice than to seek help from outside sources to protect themselves from their spouse and family (e.g., police, family protection centers, friends). Moreover, Spencer et al. (2014) discussed the potential negative consequences of help seeking outside of the family, which can also become barriers to getting help, such as irreparable harm to the marital relationship by revealing family secrets, fear of scandal, blame on women for the abuse, divorce and stigma of divorce, depriving women of custody of their children, and social isolation.

Additionally, two articles, each of which discussed the reasons why women resort to not disclose violence and remain silent (Clark et al., 2010; Al-Modallal, 2017), as resistance strategies to DV, spotted the following reasons: (1) to maintain the family unit and the use of patience with the abuser (Al-Modallal, 2017); or (2)the woman's natal family's unwillingness to provide assistance as they are not ready to take on the responsibility of women's children, thus playing a role in the women‘s misery, blaming themselves for the situation, trying to push themselves to leave their husbands, avoiding scandal, shame and jeopardizing their reputation (Clark et al., 2010).

These results give an overview of the use of resistance strategies. After a thorough reading of full-text articles, our review did not find any that clearly detailed the appropriate strategies for each form of DV or the results of the resistance on women or family, even though two studies indicated trade-off between the strategies used. Btoush and Haj-Yahia (2008) found that the preferred response for coping with marital abuse and violence was the expectation that the abused wife should change her behavior, and thus, assume responsibility to change her husband, followed by resorting to informal agents (family or community or religious figures). Battered women prefer solving their problems within the extended family, rather than resorting to divorce, because the latter will expose them to social ostracization; they are accused of not caring for their children and family, and are described as rebels. Therefore, they do not confront the husband or express a desire to divorce or separate, and do not resort to formal agents (social welfare programs, counseling, legal system) except in cases of repeated abuse and severe physical violence. Additionally, seeking help from the family is not a chosen alternative if women are exposed to the violence of an electronic dating violence, due to fear of being killed, and realizing that they would continue to experience multiple forms of abuse by their family; therefore, women report to their friends and seek counsel, or secretly turn to the police (Alsawalqa, 2021a). Alsawalqa (2021a) also confirmed that resorting to the police secretly was helpful and the best strategy, because the police are aware of the cultural and social context in Jordan and know how to maintain secrecy from the family and women's safety.

Moreover, the articles reviewed did not define which strategies are best for each form of DV according to important variables (for instance, women's social status, women's economic and educational level, age). Additionally, they did not discuss the issue of differing strategies according to the abuser's status (husband, father, brother, in-laws), and the severity of the violence, or when a woman faces more than one form of DV at the same time from one abuser or more (e.g., women may face psychological, economic, and physical violence from only her husband, or from her husband and father simultaneously). Furthermore, they did not specify the nature of the help, support, and resources that abused women receive from family, friends, or official institutions. In light of the complex relationship between the types of Jordanian women's resistance strategies and the forms of DV they are subjected to—especially in the strict patriarchal context—and between DV (particularly sexual violence) and help seeking outside the family, further research is needed for better understanding (Spencer et al., 2014).

## Discussion and future research

Our scoping review revealed the knowledge gaps in the literature about women's resistance to DV in Jordan and provides an overview and basic knowledge of their resistance strategies. The articles we considered have essential weaknesses in defining resistance strategies due to the lack of conceptual clarity of resistance, where it implicitly conceptualizes resistance as a consequence of DV and a mere means of survival. In a scoping review study, Rajah and Osborn (2020) analyzed and synthesized 74 research articles published in English-language scholarly journals between 1994 and 2017 on women's resistance to intimate partner violence, and found that resistance was undertheorized and undefined, and needs conceptual clarity.

Moreover, the conceptual ambiguity of resistance in DV research in Jordan may be due to the context of men's coercive control and patriarchal structure adopted by the hypotheses and employed in their interpretations. The link between patriarchal structure and DV, particularly in an Arab-Islamic cultural context, is significant in understanding the circumstances of Jordanian women's resistance strategies, and how they can be adapted or replaced by alternatives (Heilman et al., 2017; Alsawalqa et al., 2021), especially when the experience of battered women depends on their social position (Hayes, 2013). Jordanian society has a Bedouin-tribal, patriarchal culture, which believes that women are inferior to men, and treats them as deficient in religion and intellect. It reinforces male dominance and pressurizes men to adhere to roles and cultural ideals of manliness, which confirm social privileges and control of leadership and property for men, and encourage them to be cruel, independent, and strong, and show transgressive emotional behaviors. Therefore, Jordanian society structure establishes the relationship between men and women on the basis of coercive control and creates a context that justifies men's violence against women, and forces women to tolerate and accept it (Mango, 2017; Alsawalqa et al., 2021). Even outside the context of marital and family relations, Jordanian society prefers male employees in administrative positions even if they are unqualified, because the prevailing stereotype deems women unfit for positions of leadership and expects them to conform to socially determined positions and monotonous roles (Mango, 2017). Women in decision-making positions and public affairs (e.g., women candidates or elected women at all levels of representation [local, municipal and governorate councils, or parliament]) do not report violence in political and public life, because they fear appearing weak or incapable in the world of politics, and it negatively affects their political future, their leadership position, and support level provided by their family. Additionally, the authorities may not take a complaint of violence seriously, especially when there is no apparent physical violence (National Democratic Institute Coalition of Arab Women MPs to Combat Violence against Women, 2020). Although professional Jordanian women try to resist the dominance of the patriarchal discourse and create new discourses that reject the inferior positioning of women, they have chosen not to take an overt deliberate activist stance to implement change (Mango, 2017).

It was possible to use the context of coercive control, gender roles, female stereotypes, and the manner in which society requires men to act, in expanding the understanding of Jordanian women's resistance strategies to DV through examining the radical shift that took place in the understanding of DV which includes; understanding social reactions and legal responses to women's use of violence against a husband or a family member, the battered husband syndrome, discussing the factors of DV against women associated with husband-related factors more than women's empowerment indicators (e.g., sociocultural differences in family backgrounds, poverty, lack of harmony and understanding between spouses, jealousy), and women's ability to manipulate stereotypical features as a resistance strategy (Alsawalqa, 2021b). During marital disputes, Jordanian women resort to numerous tactics, including refusal to have sex, husband's deprivation of children, isolation, and money (e.g., if their husband is unemployed or they earn more than the husband) to respond to the man's abuse (Alsawalqa, 2021b). The lack of definition of women's resistance strategies leads to understanding DV in an undifferentiated way, without identifying each form of DV and the behaviors they involve. For instance, economic abuse against women in Jordan includes two types: (1) controlling economic resources and managing financial decisions, and (2) exploiting their economic resources (Alsawalqa, 2020, 2021c). More information is needed on contexts and forms of DV to explore strategies of resistance more clearly, which may give women the opportunity to choose the strategy that suits their circumstances to end violence.

Our results establish that the majority of Jordanian women's resistance strategies to DV were daily resistance and hidden activities, for immediate and de facto gains; among these gains, to avoid beatings, divorce, family disintegration, and to maintain economic stability. Owing to the strict patriarchal context in Jordan, women are strongly bound by traditions and cultural rules, lack all means of empowerment, and stay with an abusive husband for several reasons: the inherited social background, financial dependency, lack of family support, self-sacrifice for the sake of the children, and the adverse social consequences of a divorce (Gharaibeh and Oweis, 2009). Silence and not seeking help are the most common strategies among Jordanian women to resist DV. As for married women, their silence entails a change in their behavior and assuming responsibility to change their husband, avoidance, isolation, patience, and seeking social advice. Pain (2014) described resistance in DV situations as not always planned and strategic but faltering and unanticipated, and private and small-scale. The strategies of resistance are a mechanism of physical, economic, and existential survival (Rajah and Osborn, 2020), where women attempt to change the abuser's behavior, and challenge his perception of control, to reduce or eliminate violence (Paterson, 2009; Parker and Gielen, 2014), or to show that they are not passive or helpless (Abraham, 2005). The diversity of resistance strategies should be explored according to the nature and severity of violence, and the abuser's status (husband, father, brother, in-laws). Additionally, factors that play a critical role in women's choice of the optimal strategy (e.g., their social status, educational and economic level, their employment status, age, marital status—divorced, widow, single—and residence: rural, urban) should be investigated further. In other words, to be able to identify appropriate strategies to resist violence, it is necessary to determine who the abused woman is, who the abuser is, the motivation and severity of the violence, and the socio-cultural context of the violence. Examining resistance strategies in light of the above-mentioned factors may reduce the prevalence and consequences of DV, decipher the reasons for the failure of women's resistance to end or avoid violence, and ensure that women do not become engaged in a cycle of violence (Dasgupta, 2002; Abraham, 2005). When forces are not balanced between the parties of the abusive relationship and the strategy is inappropriate, women's resistance may become the cause of violence continuing in a perceived cycle, with its level increasing over time (Hayes, 2013). Therefore, future research should focus on forms and contexts of overt resistance, and the potential adverse consequences of hidden and overt resistance, which may reveal more about men's reactions, and thus, enable abused women to discern and plan alternative strategies.

Finally, our review indicates that future research in Jordanian women's resistance to DV should use mixed methods to reveal the context of violence, and strategies of resistance for each form of DV. Given the high incidence of DV and considering that Jordanian women resort to daily hidden resistance and its negative consequences (especially stigma, shame, deprivation of children, long-term abuse).

## Implications for practice, policy, and research

Our scoping review highlighted the lack of conceptual clarity on DV and the insufficient differentiation between its forms and the various behaviors involved in each form; without a clear research basis, policies and practices aimed at supporting women and girl's victims to DV risk being ineffective. Therefore, the understanding of Jordanian women's resistance to DV was limited to the legal response and legislative amendments that try to combat the abuse, and to provide protection, health and social care to female victims with caution, in line with the patriarchal structure and tribal culture in Jordan, which consider as a reference contributor to building social and cultural policies, which led to make the majority of women's resistance a hidden and daily activities. This type of resistance did not contribute to reducing or ending DV as much as making women enter into a cycle of endless violence, accepting and justifying the DV, and remaining in abusive relationships, Also, they fail to benefit from legal support, or access social and healthcare services which have an active role in supporting survivors of DV.

Therefore, we recommend researchers, especially Jordanians, social service practitioners, and healthcare providers, to work on recalibrating the concept of DV by considering its differentiated forms. This is necessary in light of a radical shift that took place in the understanding of DV that transcends the monocular-interpretation of the relationship between men and women based on coercive control and the victim-woman model. Future research and practice should include the battered husband syndrome, and the perspective of the man box, to understand causes and forms of DV against women more comprehensively and deeply, taking into consideration the social and cultural changes affecting Jordanian women's status, capabilities and roles. The Man Box refer to a rigid set of expectations, perceptions, and behaviors that are considered a “real man's” behavior, are defined openly by society, and have dominance over men. Men are marginalized and stigmatized when they violate the Man Box rules by not perfectly fitting the description of a “real man.” Adherence to the Man Box is one of the root causes of the frequent male violence against women, which is associated with patriarchy and ideals of masculinity that produce cultural acceptance of the use of violence, embed practices of inequality between genders, and create and reinforce a social environment conducive to DV (Edwards and Jones, 2009; Heilman et al., 2017; Alsawalqa et al., 2021). They should thus aim to understand women's responses to DV based on modern sociological and psychological theorizing of DV, This should contribute to impelling policy makers to develop plans and legislation necessary to reduce DV, directing official institutions on how to support female victims, and helping the victims determine what services and care meet their needs. In addition, policymakers should integrate in educational curricula modern social perspectives on genders and devise educational policies aimed at producing a gradual change in the current stereotypes about men's and women's roles, and in the concepts of masculinity and femininity that reinforce the gender gap. Furthermore, a more comprehensive research of DV can help healthcare and social service practitioners overcome obstacles in women's ability to access services and welfare benefits, or to seek help.

Making structural changes in a patriarchal context and introducing new cultural standards and policies is not an easy matter. It requires the cooperation of official and non-official institutions and agencies to reformulate legislation, policies, and development, educational to determine a consistent and scientifically based movement to curb DV against women and girls. There is an urgent need to conduct seminars and workshops to create awareness for women and girls in urban and rural areas in Jordan about the optimal resistance strategies against all forms of DV depending on the context of violence, their social and economic conditions, and the status of their abusers. Additionally, it is necessary to raise their awareness about their self-image, help them understand their own reasons for getting or staying married, teach them how to manage their own emotions, and brief them on what it takes to create healthy families. Women should not be the only target for this kind of education. Jordanian feminists and human rights activists, policy and decision makers, and civil society institutions must realize that men are active members of the family and society. They should direct their efforts toward educating them about the risks of DV, family dispute resolution, and increase their awareness about dealing with life pressures, and help them develop their social communication skills, relate with women in a healthy way, and learn the foundations of proper childrearing.

## Author contributions

RA and MA: conceptualization and supervision. RA and YS: methodology and project administration. RA, MA, and YS: investigation, resources, writing—review and editing, and visualization. RA: writing—original draft preparation. AA: review and editing. All authors have read and agreed to the submitted version of the manuscript.

## Conflict of interest

The authors declare that the research was conducted in the absence of any commercial or financial relationships that could be construed as a potential conflict of interest.

## Publisher's note

All claims expressed in this article are solely those of the authors and do not necessarily represent those of their affiliated organizations, or those of the publisher, the editors and the reviewers. Any product that may be evaluated in this article, or claim that may be made by its manufacturer, is not guaranteed or endorsed by the publisher.
